# Size, sounds and sex: interactions between body size and harmonic convergence signals determine mating success in *Aedes aegypti*

**DOI:** 10.1186/s13071-016-1914-6

**Published:** 2016-12-01

**Authors:** Lauren J. Cator, Zacharo Zanti

**Affiliations:** Department of Life Sciences, Grand Challenges in Ecosystems and Environment, Silwood Park, Ascot, SL5 7PY UK

**Keywords:** Mosquito, *Aedes aegypti*, Mating success, Acoustics, Harmonic convergence, Body size, Fitness

## Abstract

**Background:**

Several new mosquito control strategies will involve the release of laboratory reared males which will be required to compete with wild males for mates. Currently, the determinants of male mating success remain unclear. The presence of convergence between male and female harmonic flight tone frequencies during a mating attempt have been found to increase male mating success in the yellow fever mosquito, *Aedes aegypti.* Size has also been implicated as a factor in male mating success. Here, we investigated the relationships among body size, harmonic convergence signalling, and mating success. We predicted that harmonic convergence would be an important determinant of mating success and that large individuals would be more likely to converge.

**Methods:**

We used diet to manipulate male and female body size and then measured acoustic interactions during mating attempts between pairs of different body sizes. Additionally, we used playback experiments to measure the direct effect of size on signalling performance.

**Results:**

In live pair interactions, harmonic convergence was found to be a significant predictor of copula formation. However, we also found interactions between harmonic convergence behaviour and body size. The probability that a given male successfully formed a copula was a consequence of his size, the size of the female encountered, and whether or not they converged. While convergence appears to be predictive of mating success regardless of size, the positive effect of convergence was modulated by size combinations. In playbacks, adult body size did not affect the probability of harmonic convergence responses.

**Conclusions:**

Both body size and harmonic convergence signalling were found to be determinants of male mating success. Our results suggest that in addition to measuring convergence ability of mass release lines that the size distribution of released males may need to be adjusted to complement the size distribution of females. We also found that diet amount alone cannot be used to increase male mating success or convergence probability. A clearer understanding of convergence behaviours, their relationship to mating success, and factors influencing convergence ability would provide the groundwork for improving the mating performance of laboratory reared lines.

**Electronic supplementary material:**

The online version of this article (doi:10.1186/s13071-016-1914-6) contains supplementary material, which is available to authorized users.

## Background

Reproductive control strategies for mosquito populations offer a potentially powerful tool for controlling diseases such as malaria, dengue, Chikungunya, and Zika. These strategies encompass a wide range of operational goals. Some methods aim to decrease disease transmission by attenuating the ability of local vector populations to transmit pathogens while others focus on supressing vector populations to break the transmission cycle. These strategies utilize equally diverse mechanisms to achieve their goals, ranging from chemical sterilisation to genetic modification [1–3]. In any case, most reproductive control strategies will involve the release of laboratory reared males which will be required to successfully mate with wild females [4–6]. Some of these strategies are at advanced levels of development with published field trials indicating that some are ready for widespread deployment as part of mosquito control programs [7–9]. Both academic and industry partners have identified the need for high throughput inexpensive methods for both improving and assessing male performance [3]*.* While knowledge is accumulating, mosquito mating behaviour remains poorly understood. The success of these new innovative tools would greatly benefit from a better understanding of the basic mating biology of the organisms we are attempting to control. This is particularly true for strategies in which large numbers of males will be required for release. There are two main avenues for increasing the likelihood that released males mate with target females. The first is by increasing the number of males released and therefore the probability that wild females encounter these males. The second is by improving the competitiveness of these males in the event that there is an encounter. The advantage of focusing male performance is that it decreases the number of males that need to be released to achieve control targets while minimizing the costs associated with production.

*Aedes aegypti* is the most medically important vector of arboviruses [10, 11]. This species has been observed to mate in aerial swarms which form around the human host [12, 13]. These swarms are primarily composed of males with females entering singly to be mated. Copula formation and insemination occur in flight and the entire interaction between male and female plays out in seconds [12, 14, 15]. Until quite recently it was assumed that male-female interactions and copula formation within these swarms were essentially random with no courtship [14]. However, findings in the field of mosquito acoustics have challenged this assumption [16].

The involvement of sound in mosquito mating interactions has been known for some time [17, 18]. Recent work in *Toxorhynchites* [19]*, Aedes* [20, 21]*, Anopheles* [22, 23] and *Culex* [24] mosquitoes has demonstrated that males and females engage in acoustic signalling interactions at close range. In swarming mosquito species, males and females adjust their flight tone to approximate harmonic components of their partner’s flight tone. Further studies into this behaviour, termed “harmonic convergence”, have found that these acoustic signals are a predictor of female rejection/acceptance behaviours toward males [16]. Specifically, male-female pairs of *Ae. aegypti* which converged during the mating interaction were found to be more likely to successfully form a copula. When convergence did not occur, females were more likely to exhibit rejection behaviours towards the male [16]. The male offspring of converging pairs were both more likely to converge with a potential mate and enjoyed greater mating success [16]. The presence of these signals and their relationship to mating outcomes suggests that male acoustic signal characteristics relate to mating success. Almost all of the work on harmonic convergence to date has treated convergence as a binary response; a pair either converges or it does not [16, 19, 20, 23, 24]. In other insect signalling systems it is specific components of signals that are correlated with mating success [25–27]. Other dimensions of harmonic convergence behaviour such as the latency of response, speed of response, and duration of convergence have been left largely unexplored (for exception see [22]). Further, the importance of acoustic signals relative to other determinates of mating success remain unknown.

Body size is an easily manipulated and measured characteristic that can be used in the design and quality control of mass reared lines. Across several studies, larger female mosquitoes have been found to be more fecund [28–30], preferred by males [31], and be more likely to be inseminated in field collections [32]. The relationship between body size and male mosquito mating success is less clear. Larger males have been demonstrated to have greater capacity to produce and transfer sperm to females [33, 34]. However, in mating competition experiments both large [14, 35–37] and intermediate [38] sized males have been found to be more successful. One published study reported no relationship between body size and male mating success [39]. It should be noted that almost all of these studies have been conducted on *Anopheles* mosquitoes. The majority of the data on the role of size in male mating success available for *Aedes* mosquitoes focuses on male post-copulatory mating success [15, 33, 34].

Clarifying the role of body size in male-female mating interactions and specifically in male mating success will help inform control efforts. Different rearing conditions such as larval density, food amount, and larval nutrition yield consistent differences in adult body size [10]. The effects of body size on male survival and other traits has been incorporated into rearing designs [40]. If body size, especially body size relative to potential mates, has a significant impact on male mating success, then the effect of rearing conditions on body size should be taken into account when designing mass-rearing protocols.

Here, we explored the relationships between body size, harmonic convergence, and the outcome of mating attempts. We manipulated diet amount to generate male and female *Ae. aegypti* of different body sizes. We subjected males and females from these treatments to playback experiments using either recordings of live potential mates or computer generated tones to measure the effect of body size on convergence probability and characteristics. We then recorded acoustic interactions of live mating attempts between different male-female body size combinations. We predicted that large individuals from the high diet group would be more likely to converge and that these individuals would also enjoy higher mating success.

## Methods

### Mosquito rearing

*Ae. aegypti* eggs were taken from a colony originating from Fort Myers, Florida, USA (F6-7 generations from field material) [41]. Eggs were synchronously hatched by holding them under a vacuum for 30 min. Hatch flasks were provided a pinch of ground fish food diet (Cichlid Gold, Hikari, Kyrin Food Industries Ltd., Japan) and placed in a 27 °C incubator overnight. First-instar larvae were sorted into groups of 200 larvae/l distilled water. Two distinct feeding regimes were used to produce adults. A “high” food treatment group was provided 60 mg of diet each day (approx. 0.3 mg/larva). The “low” food treatment group was provided with 20 mg of diet each day (approx. 0.1 mg/larva). Food was supplied from day 0 to day 11 of larval development. These food amounts were determined in preliminary experiments and significantly impacted other key life history traits such as immature mortality and development times (Additional file 1: Figure S1). Pupae were individually placed in 15 ml falcon tubes plugged with cotton wool for emergence. Males and females from each treatment group from each day were transferred to sex-segregated adult cages (30 × 30 × 30 cm) and maintained on 20% sugar solution at 27 °C, 80% R.H. for 3 days.

### The role of body size on harmonic convergence to playbacks

**Fig. 1:**
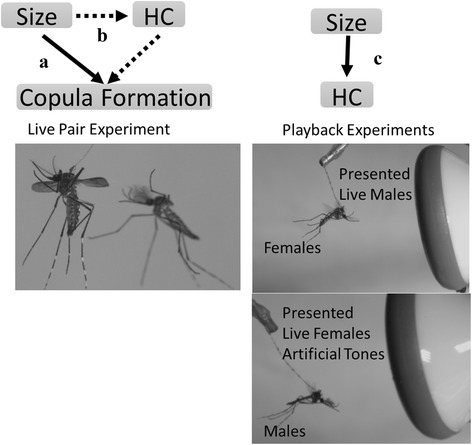
To look at the relative importance of harmonic convergence and body size we recorded interactions between live pairs of different body sizes. We examined direct effects of body size on copula formation (*Solid Line* **a**) and also whether body size indirectly affected the probability of copula formation by increasing harmonic convergence incidence (*Dashed Lines* **b**). Finally, we measured the direct effect of harmonic convergence on copula formation (*Solid Line 4*). To assess the effect of body size on harmonic convergence (*Solid Line*, **c**) we used playback experiments

The relative importance of male and female roles in harmonic convergence is unclear, but both parties appear to dynamically adjust flight tone during the interaction. In order to isolate the effect of body size on convergence behaviour we measured response of males and females from both treatments to playbacks. A single 3 to 6 day old virgin test mosquito was anesthetized on ice for 3–5 min prior to tethering [16]. Each individual was tethered to a 2–3 cm strand of human hair (LJC) using adhesive (Nailene, Pacific World Inc. Aliso Viejo, CA, USA) to fasten the tip of the hair to the dorsal anterior portion of the thorax [16]. Tethered test mosquitoes were positioned 2 cm in front of a speaker (SONY, Tokyo, Japan) [20, 22] and 2 cm above the sensitive face of a particle velocity microphone (Fig. 1, NR-21358; Knowles, Itasca, IL, U.S.A.). Natural populations of *Ae. aegypti* have been reported to mate in proximity to human hosts [12, 13]. Host stimuli were provided by the close proximity of the observer (outside of arena and less than 30 cm). All experiments were run at ambient laboratory temperature (20 ± 5 °C, 40–60% relative humidity) and conducted between 0600 and 1800 h. Once the test mosquito established flight, a panel of playback pulses representing potential mates was played to it through the speaker.

First, we investigated the responses of both large and small males and females to recordings of live potential mates (referred to as “live recordings”). Live recordings consisted of 10 s sound clips of live conspecifics in flight with 5 s breaks between each pulse. The pulses alternated between recordings of individuals from the same low diet and high diet treatments recorded in solo flight using the same experimental equipment and at similar temperature and humidity conditions. Test mosquitoes heard a total of four pulses (two from low treatment, two from high diet) played at approximately 60 dB per trial and the order of playbacks was randomised (Table 1). We recorded the responses of a total of 245 test mosquitoes to live recordings over 2 replicates.

**Table 1:** Summary of playbacks. Details of origin and frequency of stimuli used in live recording and artificial playback experiments

Live recording: female playbacks to males	
1	Low diet female
2	High diet female
3	Low diet female
4	High diet female
Live recording: male playback to females	
1	High diet male
2	Low diet male
3	High diet male
4	Low diet male
Artificial playbacks: fundamental tones played back to males (playback included this fundamental plus the next 3 harmonics)	
R1	400, 430, 460 Hz
R2	400, 430, 460, 480 Hz
R3	400, 430, 460, 480, 515, 550 Hz

To specifically investigate the effect of male size on harmonic convergence behaviour we additionally recorded male response to computer generated artificial playback mimicking females (referred to as “artificial playbacks”). Previous studies have utilised these computer generated pure tone stacks mimicking conspecifics to test male response [22]. Artificial playbacks allowed us to investigate male response to a stationary target and this type of standardized test would be useful for mass rearing programs. In this case, the playback stimulus consisted of a pure tone fundamental frequency along with the first 3 pure tone components of the harmonic stack. The harmonic components occurred at multiples of the fundamental frequency and were attenuated 5 dB at each step. The full playback was presented at approximately 60 dB. The artificial female flight tone used in playback experiments was constructed using acoustic synthesis software (Audacity, Version 2.1.1). The playback consisted of a total of eight pulses with fundamental frequencies ranging from 400 to 550 Hz (Table 1). Each pulse lasted 10 s with a 5 s break between pulses [22]. This experiment was replicated 3 times with at least 50 males recorded per treatment (318 males total over 3 replicates, Large Male (LM) = 161, Small Male (SM) = 157).

We analysed sound recordings of mosquito flight tone using Raven 1.5 (Cornell Lab of Ornithology, Ithaca, NY, USA). A harmonic convergence event was counted when any of male and female harmonic frequencies converged between the live mosquito and any of the playback frequencies. Frequencies were considered to be converging if they were within less than 4.95 Hz [20]. If we detected convergence, we then recorded the time (s) of harmonic convergence, the movement of fundamental frequency leading to convergence (ΔHz = fundamental at start of interaction- fundamental at the time of harmonic convergence measured in Hz), the duration of convergence (s), and the rate of convergence (Δ Hz/Δ s) [22]. We also noted the initial test mosquito frequency prior to playbacks, total number of separate convergence events in the playback period, and range of frequencies matched for each male.

### The role of body size on harmonic convergence and mating success in live interactions

To investigate the relative importance of male body size, female body size and harmonic convergence on copula formation, we recorded live interactions between males and live females from the different rearing conditions (Fig. 1). This resulted in four types of pairs, large female-large male (LFLM), large female-small male (LFSM), small female-large male (SFLM), and small female-small male (SFSM). Females were tethered as described above and positioned in a mating arena which consisted of a 36 cm^3^ box of transparent plastic. Once a female established flight, groups of 5 males of one body size were released into the arena for trials. We recorded the first flight tone interaction between the tethered female and a single male. We also recorded the outcome of the behavioural interaction associated with the acoustic interaction as described in [16]. A mating attempt was defined as a free flying male making physical contact with the tethered female. We classified the outcome of an attempt as a rejection or acceptance. Rejections were defined by females kicking away males, holding them away from contact with their legs, or otherwise physically preventing the contact of the genitalia. An attempt was consider successful if a copula was formed. A successful copula formation was recorded when males were able to males position themselves venter-to-venter and clasp the genitalia of the female for a minimum of 5 s. It is important to note that copula formation in *Ae. aegypti* is not necessarily indicative of sperm transfer [10, 42]. Copula formation is the end of the precopulatory phase of mating behaviour with which were concerned in this study. If no interactions occurred within 5 min we recorded a non-event. We removed the right wing of the females and all males in the trial for wing length measurements to confirm body size. This experiment was replicated 4 times (270 pairs recorded in total, totals over 4 replicates; LFLM = 77, LFSM = 72, SFLM = 61, SFSM = 60).

For live pair recordings we used the same definition of a convergence event as in playback experiments. Additionally, we recorded the movement of male and female fundamental frequency leading to convergence (ΔHz), the duration of convergence (s), and the male and female rate of convergence (Δ Hz/Δ s). We also recorded the initial female flight tone (Hz prior to male approaching) and both starting and stopping frequencies for males and females (Hz at the start of an interaction and end). Finally, we recorded the start time of the interaction in the recording (s). All acoustic analyses were completed without knowing the outcome of the interaction (copula formation or rejection) to prevent bias in interpretation of spectrograms.

### Statistical analyses

First, we confirmed that our high and low diet treatments yielded males and females of different body sizes using a General Linear Model (GLM) with wing length as the outcome variable and sex (male/female), replicate, and diet treatment (low/high) as the predictors. For artificial playback experiments, we tested the effect of male size (small/large), playback frequency (400–580 Hz), playback order and replicate on the probability of male response. The effect of sex (male/female), test mosquito size (small/large), playback size (small/large), playback order and replicate on the probability of convergence was tested in live recording playbacks. We used Generalized Linear Models (GZLM) fitted with a binary logistic regression to investigate the determinants of convergence (yes/no) and copula formation (yes/no). For live pair experiments we tested the effect of male and female treatments (small/large), harmonic convergence (yes/no), replicate, and day on the probability of successfully forming a copula. To look at the effect of convergence characteristics (rate, duration, frequency change, latency) on whether copulas were formed we ran a GZLM (binary logistic) with the parameters of harmonic convergence as predictors and copula formation as the outcome. In instances in which replicate effects were significant, we confirmed the analysis using a Generalized Linear Mixed Model with replicate as a random effect.

In all models, we tested the predictors and all interactions between these parameters. These full models were reduced through stepwise elimination of non-significant interactions and terms. The reported significance values were taken from the final model. Non-significant values are those computed in the final step prior to removal of the term from the model. We used the dispersion parameter to assess whether the assumed distribution of the error structure was appropriate. Model fits were assessed through model residuals. We confirmed that the minimal model was the best fit for the data by comparing Akaike information criterion.

## Results

### Effect of diet on adult size

Our manipulation of larval diet led to significant differences in body size in males (LM, wing length 2.33 ± 0.02 mm; SM 2.02 ± 0.02 mm; *χ* ^2^ = 147.43, *df* = 1, *P* < 0.01) and females (LF, 2.81 ± 0.02 mm; SF, 2.50 ± 0.02 mm; *χ* ^2^ = 115.04, *df* = 1, *P* < 0.01).

### The role of body size on harmonic convergence to playbacks

First, we exposed males and females to live recordings (Fig. 1a). Interestingly, females were more likely to respond to this type of playback than males (Additional file 1: Table S1; *χ* ^2^ = 26.40, *df* = 1, *P* < 0.001). There was no effect of diet treatment or playback size on whether females converged (Additional file 1: Table S1) and there was no difference in the convergence characteristics of large and small females. Males exhibited a surprisingly low response to live playbacks and only 15.32% converged with a playback frequency. Large males had a higher proportion of convergence (20.69%) compared to small males (9.43%), but this difference was not significant (Additional file 1: Table S1).

When presented artificial playbacks, 67% of males responded to at least one playback pulse. Within converging males, males responded to 24.46% of the panel of playback tones they were presented with (*n* = 214). There was no effect of the where in the playback order the frequency was given on the probability of response (Additional file 1: Table S2). There was no difference between the proportion of small and large males that converged with a playback (68% LM *vs* 66% SM). There was also no difference within responding males in the proportion of pulses they were able to converge with (24.71% LM *vs* 24.22% SM). Average convergence characteristics of male responses to playbacks can be found in Table 2. There was no effect of male treatment on male rate, change in frequency, duration or latency of response when presented with playback tones.

**Table 2:** Convergence characteristics of males responding to artificial playbacks. Sample sizes for each calculation are indicated in parentheses. The variation in sample sizes is due to some males starting the interaction in convergence (no latency or rate calculation for these males). Mean and standard error are presented

Convergence characteristic	Large males	Small males	All males
Range responded to	400–550 (205)	400–515 (189)	400–550 (394)
AP frequency responded to (Hz)	437.98 ± 2.15 (205)	438.15 ± 2.10 (189)	438.06 ± 1.50 (394)
Rate (Hz/s)	12.82 ± 2.58 (68)	10.75 ± 1.67 (73)	11.75 ± 1.50 (141)
Duration (s)	6.18 ± 0.26 (109)	5.65 ± 0.28 (104)	5.92 ± 0.19 (213)
Latency (s)	3.27 ± 0.24 (68)	3.55 ± 0.27 (73)	3.41 ± 0.18 (141)

*Abbreviations*: *AP* artificial playback, *Hz* Hertz, *s* seconds

**Fig. 2:**
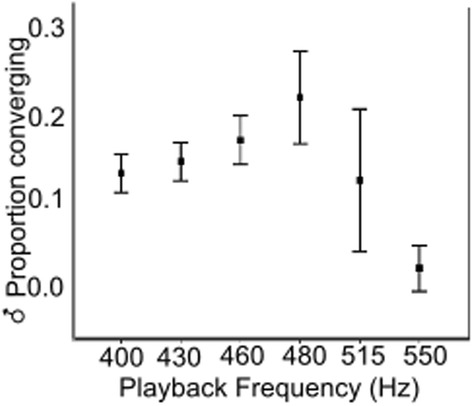
The effect of playback frequency on proportion of males converging. Proportion of males responding to artificial playbacks peaked at 480 Hz. Error bars represent ± 2 standard errors (SE)

While male body size did not affect male response, males did adjust their responses to the frequency of the stimulus they were given. Overall, males were most like to converge with tones based on a 480 Hz fundamental frequency (Fig. 2). As the replicates progressed we started including higher tones into playbacks. Consequently, we report the effect of each replicate separately in Additional file 1: Table S2. In replicate 2, males of different treatments had different responses to the frequencies presented (male treatment * playback frequency, *χ* ^2^ = 9.71, *df* = 4, *P* < 0.05). However, the difference between response to any one playback tone was not significant (Tukey’s *post-hoc* test with Bonferonni correction *P* > 0.05). In the remaining replicates, playback frequency was the only significant predictor of male response (Replicate 1, *χ* ^2^ = 8.61, *df* = 2, *P* = 0.01; Replicate 3, *χ* ^2^ = 24.86, *df* =5, *P* < 0.01). When we ran a Generalized Linear Mixed Model with replicate as a random effect, we found that only playback frequency was significant (*F* = 4.50, *P* < 0.001).

### The role of body size on harmonic convergence and mating success in live interactions

We observed 266 mating attempts across the four treatment combinations. Across all treatments, 27.82% (74/266) of interactions resulted in copula formation. These interactions started 17.26 ± 1.52 s into observations and the total acoustic interaction lasted an average of 4.18 ± 0.23 s.

Body size predicted female harmonic convergence behaviour in live pair interactions, but did not affect the likelihood that a male converged. In live pairs, large females (48.32%) were significantly more likely to converge during a mating attempt than small females (31.62%), (Additional file 1: Table S4, *χ* ^2^ = 8.28, *df* = 1, *P* = 0.004). The change in frequency made by females over the course of convergence was significantly affected by the body size of the male she interacted with (Additional file 1: Table S5, *χ* ^2^ = 4.03, *df* = 1, *P* = 0.045). Females approached by a large male altered their flight tone an average of 27.17 ± 4.52 Hz while females encountering a small male altered their flight tone an average of 15.42 ± 2.04 Hz. There was no other difference in the convergence characteristics of these pairs (Table 3). A male’s diet treatment did not affect whether he converged (Additional file 1: Table S3) and males of both treatments were more likely to converge when paired a large female.

**Table 3:** Convergence characteristics for male-female pairs from different treatments. F/M column contains the size of the female (large or small)/and size of the male (large or small) in the interaction. Male and female Δ refer to the amount of frequency change (Hz) recorded at the beginning and end of an interaction. These data are from the 109 live pairs in which convergence occurred

Male and female size	*n*	Male Δ (Hz)	Female Δ (Hz)	Latency (s)	Duration (s)	Male rate (Hz/s)	Female rate (Hz/s)
LFLM	38	63.82 ± 10.30	23.53 ± 5.73	1.71 ± 0.36	1.70 ± 0.23	73.63 ± 18.29	20.73 ± 5.21
LFSM	34	72.37 ± 11.50	14.72 ± 2.37	1.74 ± 0.29	1.11 ± 0.22	69.02 ± 14.44	24.09 ± 6.45
SFLM	18	39.40 ± 8.41	34.87 ± 7.06	1.00 ± 0.30	1.29 ± 0.28	75.50 ± 18.22	39.80 ± 7.42
SFSM	19	59.30 ± 15.81	16.65 ± 3.89	1.11 ± 0.37	1.70 ± 0.47	93.08 ± 21.92	23.84 ± 7.42

*Abbreviations*: *LFLM* large female-large male pair, *LFSM* large female-small male pair, *SFLM* small female-large male pair, *SFSM* small female-small male pair

**Fig. 3:**
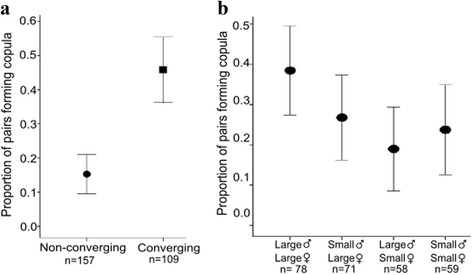
The effect of body size and harmonic convergence in copula formation. **a** Across all body size treatments the presence of a harmonic convergence event was a significant predictor of copula formation. **b** Proportion of mating interactions resulting in a successful copula formation between pairs of different body sizes. Error bars represent ± 2 standard errors (SE)

The experimental design allowed us to investigate the relative importance of convergence and body size in determining whether a pair successfully formed a copula (Fig. 1b). As reported in earlier experiments with this species [16], the presence of harmonic convergence significantly predicted a successful copula formation (Fig. 3a; Additional file 1: Table S5; *χ* ^2^ = 34.06, *df* = 1, *P* < 0.001). On average, live pair acoustic interactions lasted for 4.18 ± 0.23 s, ranging from as short as 0.11 s to as long as 20.18 s. Harmonic convergence events were found to occur in 40.98% of these live pair interactions (109/266 pairs). Our analyses of live pairs also indicated that characteristics of that convergence such as rate and duration did not affect copula formation. We compared convergence characteristics and found no difference between pairs that did or did not mate after convergence (GLMs, *P* > 0.05). Within converging pairs, none of the convergence characteristics were found to significantly predict copula formation (Additional file 1: Table S6). However, a power analysis revealed that our ability to detect such differences in these live pair interactions was relatively low (0.04–0.24) and that very large sample sizes (191–11,787 per treatment) would be required to detect any differences in convergence characteristics (Additional file 1: Table S7).

When we look at body size alone, we were able to predict copula formation in females, but surprisingly not in males. Large females were more likely to form a copula (Fig. 3b; Additional file 1: Table S8; *χ* ^2^ = 4.277, *df* =1, *P* = 0.004) with males of both body sizes. Small female pairs successfully formed a copula in 21.37% (25/117) attempts compared to large female pairs which formed copulas 32.89% of the time (49/149). Male body size alone did not significantly affect the probability that males mated (Additional file 1: Table S8).

**Fig. 4:**
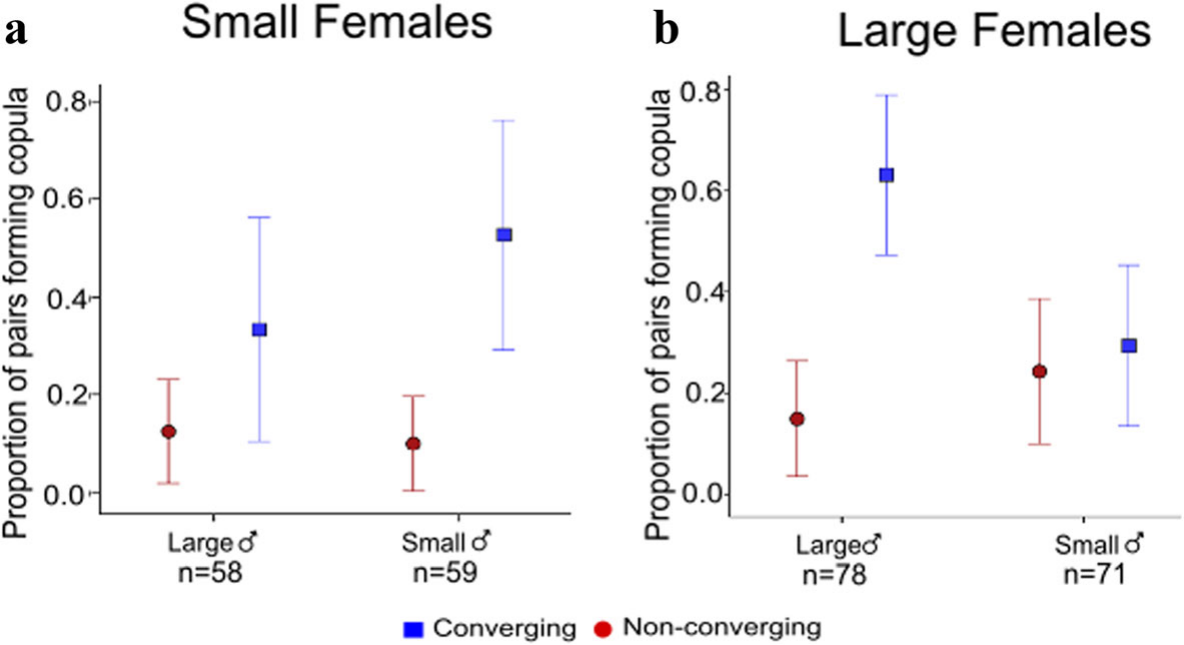
The effect of harmonic convergence across body size combinations. Proportion of copulas formed by converging (*blue, square*) and non-converging (*red, circle*) of different body sizes in live pair interactions. (*n* = 266 pairs). **a** Copulas involving small females. The presence of a convergence event increased the probability of copula formation for males of both body sizes. (*n* = 117 pairs). **b** Copulas involving large females. An interaction between male size and presence of harmonic convergence increased the likelihood of male mating success when approaching large females (*n* = 149 pairs). Error bars represent ± 2 standard errors (SE)

When we took into account both harmonic convergence and body size we found that the size of a male, the size of the female he encountered, and whether or not they converged all determine copula formation. (Fig. 4; Additional file 1: Table S5; male treatment * female treatment * harmonic convergence, *χ* ^2^ = 16.61, *df* = 6, *P* = 0.01). Harmonic convergence increased the probability of copula formation for pairs of the same size more than pairs of differing size (47.15% *vs* 12.59%). This apparent assortative effect is driven by interactions between large individuals. In pairs involving a small female, the presence of a convergence event was the single significant determinant of copula formation (Fig. 4a; *χ* ^2^ = 17.75, *df* = 1, *P* < 0.001). In contrast, in pairs that involved a large female, males which converged with their mate and were large had a significantly higher probability of successfully forming a copula (Fig. 4b, *χ* ^2^ = 12.13, *df* = 2, *P* = 0.002). Pairwise comparisons revealed that, for large females, only those large males with which they converged had a significantly higher probability of copula formation (Tukey’s *post-hoc* with Bonferroni correction, *P* < 0.05). In other words, when encountering a large female, the positive effect of convergence on copula formation was greater for large males.

## Discussion

Both size and harmonic convergence signals were important in determining whether a given male formed a copula. There was no direct relationship between male size and convergence behaviour and the proportion of pairs converging at harmonic frequencies did not vary with male-female body size combination. Instead, the degree of increase in mating success associated with the presence of harmonic convergence was dependent on male size and the size of the female he attempted to mate with.

On average harmonic convergence increases mating success between pairs of the same size more than pairs of different sizes (Fig. 4). However, this effect is driven by interactions between large females and large males rather than a general assortative relationship. It was only when we took into account the combined effects of harmonic convergence signals and body size that we were able to explain variation in the probability of male copula formation. For pairs involving large females, both male size and convergence were important. Specifically, large males approaching a large female and converging were more likely to form a copula compared to all small males and large males that did not converge. For pairs involving small females the only significant factor in determining copula formation was the presence of a harmonic convergence event. Male size was not important in these interactions (Fig. 4).

While male mating success with all females is of interest, the ability of males to mate with large females is particularly important. These large females would be predicted to have greater lifetime fecundity [28–30, 32, 43]. Thus, both male size and harmonic convergence ability were an important determinant of male mating success for the subset of the female mosquito population with the greatest fecundity. In reproductive strategies aiming at decreasing vector populations, curtailing the reproduction of these females would facilitate population control. In strategies aimed at replacing the population, these females would produce more offspring carrying the trait of interest.

Our results suggest two key things about the determinants of male mating success and how large-scale rearing programs could assess and improve male *Aedes* mating potential. First, the factors determining variation in male mating success appear to be more complicated than “bigger is better”. Trends in the data show that large males attempting to mate with large females had higher mating success, but mating success of these males was lower when attempting to mate with small females (Fig. 3b). This contrasts with the majority of studies in *Anopheles* mosquitoes which find that larger males are more likely to form a copula in field swarms [35–37]. Consequently, because females from target field populations will contain a mix of body sizes, release males may perform more effectively if reared to have a range of body sizes reflecting this distribution.

In these experiments we focused on isolated interactions between a single male and female. This moment of copula formation is a key event in mating, but is certainly not the only determinant of male mating success in this species. Males of different sizes may experience differential encounter probabilities with females due either to differences in survivorship or swarming activity (Lang and Igodube et al. in prep). In this study we focused on pre-copulatory behaviour and so did not measure insemination success. Several studies have demonstrated important post-copulatory differences in large and small males [15, 34, 44]. These elements may ultimately be most important for determining overall male fitness in this species and future experiments could tie these different elements together. Our results here highlight the need for expanded studies on the role of male size in pre-copulatory mating behaviour in *Aedes*, similar to those conducted for *Anopheles*.

Second, our results strongly suggest that maintaining or enhancing a release line’s ability to converge with potential mates will be important for maximizing their efficiency. We found that the presence or absence of a harmonic convergence event was the single most significant predictor of copula formation across males. While increasing male body size could marginally improve male mating probability (approximately 5% increase, Fig. 3b), ensuring convergence ability would offer a substantial benefit (approximately 30% increase, Fig. 3a). Thus, further work on the exact characteristics, function, and determinants of harmonic convergence would be useful in the creation of mass-reared lines.

A trade-off between frequency and temporal resolution is an implicit characteristic of Fourier analysis [45]. This makes analyses of harmonic convergence interactions which involve rapid modulation in frequency particularly difficult. We found no effect of specific signal parameters such as duration or rate on copula formation. There is a possibility that we were unable to measure variation in these parameters with sufficient accuracy to detect differences. Even with a fairly basic presence or absence test we can see that these acoustic interactions have important implications for male mating success. Further development of these recording and signal processing tools will enhance our understanding of this biological phenomenon [21].

The exact function of the harmonic convergence signals is still unresolved. The mid-air acrobatics required in mosquito mating are not negligible and males and females may use convergence to coordinate the movements required for copula formation. There are several examples of these types of signals in the final stages of courtship which function to coordinate mating after a choice has been made [46, 47]. Interestingly, harmonic convergence behaviours were recently reported in midges which also mate in flight [48]. Males in converging pairs may have been accepted by females using other cues earlier in the mating attempt. The relationship between copula formation and convergence may therefore be a consequence of this acceptance rather than a signal that females use to inform acceptance decisions. Future work including detailed analyses of free flight interactions could help provide insight into these questions.

Alternatively, females may use harmonic convergence as a signal of male quality. The information content of these signals can provide insight into the potential determinants of harmonic convergence ability and provide information about how to rear mass release males to maximize convergence potential. In some instances, acoustic courtship signals indicate high levels of energetic reserves. If this is the case with harmonic convergence then increasing the amount of larval diet could increase the probability that males converge. Similar manipulations of diet have been used in other male insect release programs to improve male signalling performance [49]. However, in both live pairs and playback experiments, we found no effect of diet on male harmonic convergence responses. Both large and small males are equally likely to converge. The diet treatments we used were significant enough to cause changes in male and female body size, survival, and development time so it would be expected that these diets would be sufficient to alter signalling ability.

Previous work suggested that both harmonic convergence signalling ability and mating success are heritable [16]. In many respects, mosquito swarms resemble lek mating systems. Male *Ae. aegypti* do not offer resources or parental care to females. In these types of mating systems, females often use behavioural traits as opposed to morphological traits to distinguish between the genetic benefits offered by potential mates [47]. These behavioural traits are more varied than morphological traits such as body size, typically require the coordination of several physiological systems and provide females with an overall “diagnostic” of male quality [47]. The exact mechanisms of flight tone production, perception and processing in mosquitoes is currently being investigated [19, 20, 50–52], but are likely complex, involving integration of information from many neurons and the indirect flight muscles.

While the mechanics of sound reception were not our focus here, the response of females to playbacks is intriguing given that previous work indicates that females are not able to perceive these frequencies as well as males. The incorporation of paired behaviour and physiological experiments may provide additional insight into the currently expanding field of mosquito audition and improve our understanding of the role of acoustics signalling in mosquito mate choice.

Our results support the idea that harmonic convergence is a dynamic process. Both males and females adjusted signal responses depending on the frequency of potential mates (female frequency change in live pairs and male convergence probability in playbacks). Interestingly, males were much more likely to converge with artificial playbacks than live recordings of females. This may be because the artificial tones were held at a constant frequency that was easier for males to converge with or because the flight tones of live females which were not hearing males were variable in a way that was not conducive to convergence. A male’s ability to respond dynamically to female flight tone may allow females to assess heritable male traits that will improve offspring success. For example, males with the ability to converge may produce offspring with superior flight reflexes. These reflexes may be important for offspring survival in other contexts. If harmonic convergence ability has heritable genetic determinants then this trait should be specifically tested for in the assessment of release lines.

## Conclusions

There are several examples of reproductive control programs that have successfully increased efficacy by taking into account the mating biology of target organisms [49]. Despite intense study of *Ae. aegypti* as a disease vector, our understanding of the mechanisms and traits involved in male mating success remain incomplete. Our results suggest that both environmentally determined traits such as body size and heritable traits such as is potentially the case with harmonic convergence ability contribute to male mating success. Further investigation of these signals and their role in mating behaviour will improve our understanding of male mating success, mechanisms of female choice, and mosquito mating biology. Ultimately, this improved understanding of mosquito biology will improve our ability to improve the fitness of released males and implement successful reproductive control programs.

## Additional file

Additional file 1: Figure S1, Tables S1-S8. This file contains data the effect of treatments on immature development and mortality and details of all model outputs. (DOCX 130 kb)

## Abbreviations

GLM: General linear model
GZLM: Generalized linear model
LF: Large female
LFLM: Large female-large male pair
LFSM: Large female-small male pair
LM: Large male
SF: Small female
SFLM: Small female-large male pair
SFSM: Small female-small male pair
SM: Small male

## References

[1] Caragata EP, Dutra HLC, Moreira LA (2016). Exploiting intimate relationships: controlling mosquito-transmitted disease with *Wolbachia*. Trends Parasitol.

[2] O’Brochta DA, Takken W, Scott TW (2003). Transgenic mosquito: the state of the art. Ecological aspects for application of genetically modified mosquitoes.

[3] Alphey L, Benedict M, Bellini R, Clark GG, Dame DA Service MW (2010). Sterile-insect methods for control of mosquito-borne diseases: an analysis. Vector Borne Zoontic Dis.

[4] Alphey L (2014). Genetic control of mosquitoes. Annu Rev Entomol.

[5] Burt A (2014). Heritable strategies for controlling insect vectors of disease. Philos Trans R Soc Lond B.

[6] Frentiu FD, Zakir T, Walker T, Popovici J, Pyke AT, van den Hurk A (2014). Limited dengue virus replication in field-collected *Aedes aegypti* mosquitoes infected with *Wolbachia*. PLoS Negl Trop Dis.

[7] Harris A, McKemey A, Nimmo D, Curtis Z, Black I, Morgan S (2012). Successful suppresion of a field mosquito population by sustained release of engineered male mosquitoes. Nat Biotechnol.

[8] Harris A, Nimmo D, McKemey A, Kelly N, Scaife S, Donnelly C (2011). Field performance of engineered male mosquitoes. Nat Biotechnol.

[9] Lacroix R, McKemey A, Raduan N, Kwee Wee L, Hong Ming W, Guat Ney T (2012). Open field release of genetically engineered sterile male *Aedes aegypti* in Malaysia. PLoS ONE.

[10] Clements AN (1992). The biology of mosquitoes, volume 2 sensory reception and behaviour.

[11] CDC Surveillance and Control of *Aedes aegypti* and *Aedes albopictus* in the United States. 2016

[12] Cator LJ, Arthur BJ, Ponlawat A, Harrington LC (2011). Behavioral observations and sound recordings of free-flight mating swarms of *Ae. aegypti* (Diptera: Culicidae) in Thailand. J Med Entomol.

[13] Hartberg W (1970). Observations on the mating behavior of *Aedes aegypti* in nature. Bull World Health Organ.

[14] Yuval B (2006). Mating systems of blood-feeding flies. Annu Rev Entomol.

[15] Helinski MEH, Harrington LC (2011). Male mating history and body size influence female fecundity and longevity of the dengue vector *Aedes aegypti*. J Med Entomol.

[16] Cator LJ, Harrington LC (2011). The harmonic convergence of fathers predicts the mating success of sons in *Aedes aegypti*. Anim Behav.

[17] Mayer AM (1874). Experiments on the supposed auditory apparatus of the mosquito. Am Nat.

[18] Roth LM (1948). A study of mosquito behaviour: an experimental laboratory study of the sexual behaviour of *Aedes aegypti* (Linnaeus). Am Midl Nat.

[19] Gibson G, Russell I (2006). Flying in tune: sexual recognition in mosquitoes. Curr Biol.

[20] Cator LJ, Arthur BJ, Harrington LC, Hoy RR (2009). Harmonic convergence in the love songs of the dengue vector mosquito. Science.

[21] Aldersley A, Champneys A, Homer M, Robert D (2016). Quantitative analysis of harmonic convergence in mosquito auditory interactions. J R Soc Interface.

[22] Cator LJ, Ng’Habi KR, Hoy RR, Harrington LC (2010). Sizing up a mate: variation in production and response to acoustic signals in *Anopheles gambiae*. Behav Ecol.

[23] Pennetier C, Warren B, Dabiré KR, Russell IJ, Gibson G (2010). “Singing on the wing” as a mechanism for species recognition in the malarial mosquito *Anopheles gambiae*. Curr Biol.

[24] Warren B, Gibson G, Russell IJ (2009). Sex recognition through midflight mating duets in *Culex* mosquitoes is mediated by acoustic distortion. Curr Biol.

[25] Hedrick AV (1986). Female preferences for male calling bout duration in a field cricket. Behav Ecol Sociobiol.

[26] Gray DA (1997). Female house crickets, *Acheta domesticus*, prefer the chirps of large males. Anim Behav.

[27] Ritchie MG, Saarikettu M, Livingstone S, Hoikkala A (2001). Characterization of female preference functions for *Drosophila montana* courtship song and a test of the temperature coupling hypothesis. Evolution.

[28] Packer MJ, Corbet PS (1989). Size variation and reproductive success of female *Aedes punctor* (Diptera: Culicidae). Ecol Entomol.

[29] Briegel H (1990). Metabolic relationship between female body size, reserves, and fecundity of *Aedes aegypti*. J Insect Physiol.

[30] Renshaw M, Service MW, Birley MH (1994). Size variation and reproductive success in the mosquito *Aedes cantans*. Med Vet Entomol.

[31] Okanda FM, Dao A, Njiru BN, Arija J, Akelo HA, Touré Y (2002). Behavioural determinants of gene flow in malaria vector populations: *Anopheles gambiae* males select large females as mates. Malar J.

[32] Ameneshewa B, Service MW (1996). The relationship between female body size and survival rate of the malaria vector *Anopheles arabiensis* in Ethiopia. Med Vet Entomol.

[33] Ponlawat A, Harrington L (2009). Factors associated with male mating success of the dengue vector mosquito, *Aedes aegypti*. Am J Trop Med Hyg.

[34] Ponlawat A, Harrington LC (2007). Age and body size influence male sperm capacity of the dengue vector *Aedes aegypti* (Diptera: Culicidae). J Med Entomol.

[35] Gary RE, Cannon JW, Foster WA (2009). Effect of sugar on male *Anopheles gambiae* mating performance, as modified by temperature, space, and body size. Parasit Vectors.

[36] Sawadogo SP, Diabaté A, Toé HK, Sanon A, Lefevre T, Baldet T (2013). Effects of age and size on *Anopheles gambiae* ss male mosquito mating success. J Med Entomol.

[37] Maïga H, Dabiré RK, Lehmann T, Tripet F, Diabaté A (2012). Variation in energy reserves and role of body size in the mating system of *Anopheles gambiae*. J Vector Ecol.

[38] Ng’habi KR, Huho BJ, Nkwengulila G, Killeen GF, Knols BGJ, Ferguson HM (2008). Sexual selection in mosquito swarms: may the best man lose?. Anim Behav.

[39] Charlwood JD, Pinto J, Sousa CA, Ferreira C, Do Rosário VE (2002). Male size does not affect mating success (of *Anopheles gambiae* in São Tomé). Med Vet Entomol.

[40] Bargielowski I, Nimmo D, Alphey L, Koella JC (2011). Comparison of life history characteristics of the genetically modified OX513A line and a wild type strain of *Aedes aegypti*. PLoS ONE.

[41] Bargielowski IE, Lounibos LP, Carrasquilla MC (2013). Evolution of resistance to satyrization through reproductive character displacement in populations of invasive dengue vectors. PNAS.

[42] Gwadz RW, Craig GB, Hickey W (1971). Female sexual behavior as the mechanism rendering *Aedes aegypti* refractory to insemination. Biol Bull.

[43] Lyimo EO, Takken W (1993). Effects of adult body size on fecundity and the pre-gravid rate of *Anopheles gambiae* females in Tanzania. Med Vet Entomol.

[44] Helinski MEH, Deewatthanawong P, Sirot LK, Wolfner MF, Harrington LC (2012). Duration and dose-dependency of female sexual receptivity responses to seminal fluid proteins in *Aedes albopictus* and *Ae. aegypti* mosquitoes. J Insect Physiol.

[45] Chatfield C, Chatfield C (2004). Simple descriptive techniques. The analysis of time series: an introduction.

[46] Dawkins MS, Guilford T (1994). Design of an intention signal in the Bluehead Wrasse (*Thalassoma bifasciatum*). Proc R Soc Lond B Biol Sci.

[47] Bradbury JW, Vehrencamp SL (2004). Principles of animal communication.

[48] de Silva P, Nutter B, Bernal XE (2015). Use of acoustic signals in mating in an eavesdropping frog-biting midge. Anim Behav.

[49] Itô Y, Yamamura K, Dyck VA, Hendrichs J, Robinson AS (2005). Role of population and behavioural ecology in the sterile insect technique. Sterile insect technique.

[50] Simões PMV, Ingham RA, Gibson G, Russell IJ (2016). A role for acoustic distortion in novel rapid frequency modulation behaviour in free-flying male mosquitoes. J Exp Biol.

[51] Arthur BJ, Wyttenbach RA, Harrington LC, Hoy RR (2010). Neural responses to one- and two-tone stimuli in the hearing organ of the dengue vector mosquito. J Exp Biol.

[52] Lapshin DN, Vorontsov DD (2013). Frequency tuning of individual auditory receptors in female mosquitoes (Diptera, Culicidae). J Insect Physiol.

