# Supplementary material for: Size, sounds and sex: interactions between body size and harmonic convergence signals determine mating success in Aedes aegypti
Source: Parasit Vectors. 2016 Dec 1;9:622. doi: 10.1186/s13071-016-1914-6 (PMC5133739; doi:10.1186/s13071-016-1914-6)
Supplement: Additional file 1: Figure S1, Tables S1-S8. — This file contains data the effect of treatments on immature development and mortality and details of all model outputs. (DOCX 130 kb) [file 13071_2016_1914_MOESM1_ESM.docx]

**Additional file 1**


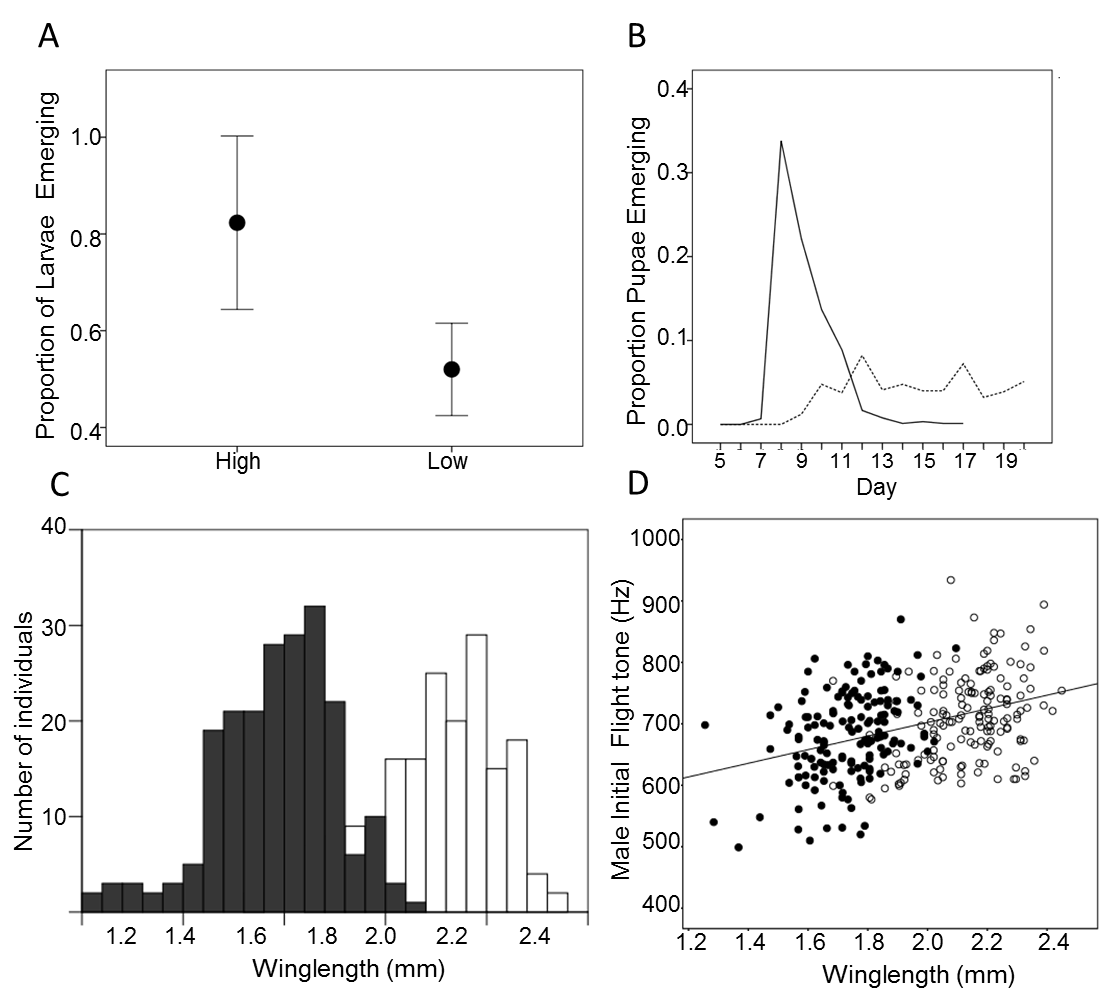


**Figure S1:** **Effect of larval diet on immature development, body size, and flight tone.** A. Individuals fed at “High” (0.3mg/larva/day) diet exhibited a higher proportion of larvae surviving to emerge as adults than those fed a “Low” diet (0.1mg/larva/day). B. High diet individuals (black solid line) emerged from pupae to adults earlier and more synchronously than Low diet indviduals (grey dotted line). C. Individuals emerging from the different treatment conditions had distinctly different winglength distributions.Grey bars correspond to low diet individuals and white bars correspond to high diet individuals. D. Male flight tone was still found to be positively correlated with wing length. Black circles low diet males and white circle are low diet males.

**Table S1**. The effect of treatment (diet, which determined body size), sex, replicate, and playback size on whether test mosquitoes responded to live recordings. Non- significant P values are from the step prior to removal. Significant p values are from the minimal significant model.

| **Parameter** | **χ^2^** | **df** | **P** |
| --- | --- | --- | --- |
| Treatment | 2.23 | 1 | 0.14 |
| **Sex** | **26.40** | **1** | **<0.001** |
| Playback Order | **4.82** | **5** | **0.44** |
| Playback Size | 0.00 | 2 | 1.00 |
| Replicate | 0.51 | 1 | 0.48 |
| Sex x Treatment | 2.25 | 1 | 0.13 |
| Playback Size x Treatment | 0.11 | 1 | 0.74 |
| Treatment x Replicate | 0.53 | 1 | 0.47 |
| Sex x Playback Size | 0.28 | 1 | 0.60 |
| Sex x Replicate | 0.41 | 1 | 0.52 |
| Playback Size x Replicate | 1.19 | 1 | 0.28 |
| Treatment x Sex x Playback Size | 0.45 | 1 | 0.50 |
| Treatment x Sex x Replicate | 0.24 | 1 | 0.62 |
| Treatment x Playback Size x Replicate | 0.52 | 1 | 0.47 |
| Sex x Playback Size x Replicate | 0.17 | 1 | 0.68 |
| Treatment x Sex x Playback Size x Replicate | 0.02 | 1 | 0.89 |

**Table S2.** Predictors of male convergence (yes/no) to artificial playbacks broken down by replicate (Table S1).

|  | **Parameter** | **χ2** | **df** | **P** |
| --- | --- | --- | --- | --- |
| 1 | Male Treatment | 0.64 | 1 | 0.42 |
|  | **Playback Frequency** | **8.67** | **2** | **0.01** |
|  | Position in Order | 12.37 | 9 | 0.19 |
|  | Male Treatment x Playback Frequency | 4.93 | 2 | 0.09 |
| 2 | Male Treatment | 1.12 | 1 | 0.29 |
|  | Playback Frequency | 4.76 | 3 | 0.19 |
|  | Position in Order | 5.89 | 6 | 0.44 |
|  | **Male Treatment x Playback Frequency** | **9.71** | **4** | **<0.05** |
| 3 | Male Treatment | 0.15 | 1 | 0.70 |
|  | **Playback Frequency** | **24.86** | **5** | **<0.01** |
|  | Position in Order | 3.84 | 8 | 0.55 |
|  | Male Treatment x Playback Frequency | 2.59 | 5 | 0.76 |

**Table S3**: Test of Model Effect for binary logistic regression for effects of replicate, male treatment, female treatment, and their interactions on harmonic convergence presence/absence. Non-significant P-values are from the step prior to removal. Significant P-values are from the minimal significant model

| **Parameter** | **χ2** | **df** | **P** |
| --- | --- | --- | --- |
| Replicate | 6.88 | 3 | 0.08 |
| **Female Treatment** | **8.26** | **1** | **<0.01** |
| Male Treatment | 0.02 | 1 | 0.87 |
| Replicate x Female Treatment | 7.62 | 3 | 0.06 |
| Replicate x Male Treatment | 2.81 | 3 | 0.42 |
| Female x Male Treatment | 0.05 | 1 | 0.83 |
| Replicate x Female Treatment x Male Treatment | 2.99 | 3 | 0.39 |

**Table S4:** Predictors of female frequency change. Females interacting with large males alter their flight tone more over the course of the interaction than females presented with small males.

| **Parameter** | **χ2** | **df** | **P** |
| --- | --- | --- | --- |
| Female Treatment | 3.25 | 1 | 0.07 |
| **Male Treatment** | **4.03** | **1** | **<0.05 (0.045)** |
| Replicate | 2.02 | 3 | 0.57 |
| Female Treatment x Male Treatment | 1.45 | 1 | 0.23 |
| Female Treatment x Replicate | 3.14 | 3 | 0.37 |
| Male Treatment x Replicate | 0.75 | 3 | 0.86 |
| Male Treatment x Female Treatment x Replicate | 1.90 | 3 | 0.59 |

**Table S5**: Test of Model Effect for binary logistic regression for effects of replicate, male treatment, female treatment, convergence presence/absence, and their interactions on the formation of a copula. Non-significant P-values are from the step prior to removal. Significant P-values are from the minimal significant model.

| **Parameter** | **χ2** | **df** | **P** |
| --- | --- | --- | --- |
| **Convergence** | **34.06** | **1** | **0.00** |
| **Replicate** | **12.77** | **3** | **0.01** |
| Female Treatment | 0.95 | 1 | 0.33 |
| Male Treatment | 0.18 | 1 | 0.67 |
| Convergence x Replicate | 3.13 | 3 | 0.37 |
| Male Treatment x Female Treatment | 4.53 | 2 | 0.10 |
| Female Treatment x Convergence | 0.34 | 1 | 0.56 |
| Female Treatment x Replicate | 2.08 | 3 | 0.56 |
| Male Treatment x Convergence | 2.10 | 1 | 0.15 |
| Male Treatment x Replicate | 3.14 | 3 | 0.37 |
| **Male treatment x Female treatment x Convergence** | **16.61** | **6** | **0.01** |
| Female Treatment x Male Treatment x Replicate | 3.69 | 3 | 0.30 |
| Female Treatment x Convergence x Replicate | 1.03 | 3 | 0.79 |
| Male Treatment x Convergence x Replicate | 1.10 | 3 | 0.78 |
| Female Treatment x male treatment x Convergence x Replicate | 3.73 | 3 | 0.29 |

**Table S6**: Test of Model Effect for binary logistic regression for effects of replicate, duration, latency, male frequency change, female frequency change, male rate, and female rate on copula formation. Non-significant P-values are from the step prior to removal.

| **Parameter** | **χ2** | **df** | **P** |
| --- | --- | --- | --- |
| Latency | 0.43 | 1 | 0.51 |
| Duration | 2.95 | 1 | 0.09 |
| Female Change | 2.34 | 1 | 0.13 |
| Male Change | 2.40 | 1 | 0.12 |
| Female Rate | 2.15 | 1 | 0.14 |
| Male Rate | 2.42 | 1 | 0.12 |
| Replicate | 1.36 | 1 | 0.97 |

**Table S7**: Power analysis for convergence characteristics from pairs which did or did not form a copula. Test based on two tailed t-test for differences between copula and non-copula forming pairs. Averages are untransformed. Power and sample size calculations were made using the transformed data for normality (Log(Latency), Log(Duration), Log (Change Female), Log (Female Rate), Log (Change Male), Sq Rt (Male Rate)).

| Outcome | Latency (s) | Duration(s) | Change in Female Frequency (Hz) | Female Rate (Hz/s) | Change in Male Frequency (Hz) | Male Rate (Hz/s) |
| --- | --- | --- | --- | --- | --- | --- |
| Copula formed | 1.52 ± 0.30 [45] | 1.68 ± 0.20 [50] | 20.49 ± 3.11 [50] | 27.62 ± 6.25 [45] | 56.85 ± 8.35 [50] | 84.66 ± 15.45 [45] |
| No Copula | 1.48 ± 0.20 [56] | 1.25 ± 0.20 [59] | 22.27 ± 4.00 [59] | 23.61 ± 3.59 [54] | 65.75 ± 8.46 [59] | 67.74 ± 10.43 [54] |
| Power | 0.04 | 0.16 | 0.23 | 0.24 | 0.12 | 0.06 |
| Sample Size required for 0.8 Power | 11787 | 428 | 228 | 191 | 615 | 2136 |

**Table S8**: Test of Model Effect for binary logistic regression for effects of replicate, male treatment, female treatment, and their interactions on the formation of a copula. Non-significant P-values are from the step prior to removal. Significant P-values are from the minimal significant model

| **Parameter** | **χ2** | **df** | **P** |
| --- | --- | --- | --- |
| Replicate | 6.429 | 3 | 0.09 |
| **Female Treatment** | **4.277** | **1** | **0.04** |
| Male Treatment | 0.712 | 1 | 0.40 |
| Replicate x Female Treatment | 2.153 | 3 | 0.54 |
| Replicate x Male Treatment | 3.389 | 3 | 0.34 |
| Female Treatment x Male Treatment | 2.345 | 1 | 0.13 |
| Replicate x Female Treatment x Male Treatment | 1.274 | 3 | 0.74 |
